# Resveratrol as a Dual MAPK/STAT3 Inhibitor in Glioblastoma: Mutation-Dependent Therapeutic Efficacy

**DOI:** 10.3390/life16050772

**Published:** 2026-05-04

**Authors:** Aziz Ullah, Mengjie Li, Mohammad Abdullah Aljasir, Sajjad Ahmad, Chuanchun Han

**Affiliations:** 1 Institute of Cancer Stem Cell, Dalian Medical University, 9 West Section, Lvshun South Road, Dalian 116044, China; lmj0419@outlook.com (M.L.); hanzc@dmu.edu.cn (C.H.); 2 Department of Medical Laboratories, College of Applied Medical Sciences, Qassim University, Buraydah 51431, Saudi Arabia; 3 Department of Health and Biological Sciences, Abasyn University, Peshawar 25000, Pakistan; sajjad.ahmad@abasyn.edu.pk

**Keywords:** glioblastoma, BRAFV600E, resveratrol, dabrafenib, trametinib, MAPK, STAT3

## Abstract

**Background**: Glioblastoma multiforme (GBM) is the most aggressive primary brain tumor with limited treatment options. Tumors harboring the BRAF^V600E^ mutation exhibit aggressive behavior and present therapeutic challenges. Although dabrafenib/trametinib (D+T) target the BRAF/MAPK pathway and show efficacy in BRAF^V600E^ mutant melanoma, their effectiveness against GBM remains unclear. RES demonstrates anti-GBM activity through the inhibition of multiple signaling pathways. This study evaluated the therapeutic potential of RES either in monotherapy or in combination with D+T in GBM cells with and without the BRAF^V600E^ mutation. **Methods**: BRAF^V600E^ mutational status was confirmed in LN428 and U251 GBM cell lines using Sanger sequencing. Cell proliferation and viability was assessed by CCK-8, EdU assay and Calcein AM/PI staining, cell morphology by H&E staining, cell migration by Transwell assay, and apoptosis by TUNEL assay. The protein expressions of BRAF, pERK, and pSTAT3 were analyzed by Western blot, immunocytochemistry (ICC), and immunofluorescence (IF) following treatment with RES, D+T, or their combination. Statistical significance was determined using one-way ANOVA followed by Dunnett’s post hoc test with *p* < 0.05. **Results**: Sanger sequencing confirmed the presence of the BRAF^V600E^ mutation in the LN428 cells and its absence in the U251 cells. In the BRAF^V600E^ mutant LN428 cells, neither RES, D+T, nor their combination inhibited cell proliferation or migration, nor did they induce apoptosis. In contrast, RES monotherapy significantly suppressed proliferation, reduced migration, and induced apoptosis in the wild-type U251 cells, while D+T showed minimal inhibitory effects in both cell lines. Western blotting, ICC, and IF analyses revealed that RES significantly downregulated both pERK and pSTAT3 expression in the U251 cells but failed to produce similar effects in the LN428 cells. Notably, D+T treatment induced marked upregulation of pSTAT3 in both cell lines, which was effectively reversed by RES treatment in the U251 cells but not in the LN428 cells. **Conclusions**: RES selectively suppressed the MAPK and STAT3 signaling pathway in the BRAF wild-type U251 cells, while demonstrating no significant inhibitory effects in the BRAF mutant LN428 cells. This differential response indicates that mutational background governs MAPK/STAT3 pathway regulation, positioning RES as a promising dual-pathway inhibitor in mutation-stratified GBM therapeutics.

## 1. Introduction

GBM is the most prevalent form of primary brain cancer, constituting approximately 50% of malignant brain tumors. Patients diagnosed with GBM face a challenging prognosis, with an average survival duration of 14–15 months [1]. Currently, the standard of care involves surgery, radiation therapy, and temozolomide (TMZ)-based chemotherapy; however, these approaches yield limited overall survival (OS) rates [2]. TMZ being the most frequently utilized chemotherapy for GBM, it is challenged by resistance and significant side effects such as infertility and bone marrow suppression [3]. Furthermore, continuous TMZ dosing can result in secondary drug resistance, tumor recurrence, and increased patient mortality [4]. The ineffectiveness of available therapies results from multiple factors, including the blood–tumor barrier, blood–brain barrier, glioma stem-like cells, and GBM genetic heterogeneity [2]. An in-depth exploration of the molecular processes underlying GBM development and effective treatment strategies is critical for identifying novel therapeutic targets and increasing patient survival rates.

V-raf murine sarcoma viral oncogene homolog B (BRAF) is a serine/threonine protein kinase crucial for regulating cell differentiation and proliferation through the initiation of the highly conserved mitogen-activated protein kinase (MAPK) pathway [5]. Moreover, point or fusion mutations in the BRAF gene can lead to excessive activation of the MAPK signaling pathway, promoting uncontrolled cellular proliferation, resistance to apoptosis, and contributing to cancer development [5,6]. A common alteration in the BRAF gene occurs at codon 600 in exon 15, where a nucleotide transversion at residue 1799 (T1799A) leads to an amino acid substitution from valine to glutamic acid (V600E), resulting in a highly active constitutive kinase [7,8]. BRAF^V600E^ represents the most frequent BRAF alteration, accounting for more than 90% of all BRAF mutations, and is generally associated with poor patient prognosis [8,9,10]. Notably, BRAF^V600E^ mutation has been identified in GBM with a reported prevalence ranging between 3 and 6% [11,12]. Nonetheless, data concerning the prognostic significance and therapeutic implications of the BRAF^V600E^ mutation in GBMs are still limited [13]. Currently, D+T are used to treat advanced melanomas with BRAF^V600E^ [14], and the synergistic use of D+T can effectively inhibit the MAPK pathway by suppressing the proliferation and viability of BRAF^V600E^-harboring cells [15]. However, the D+T combination may result in a heightened incidence of side events owing to their double toxicities [16], and drug resistance after repeated exposure [17] emphasized the necessity of exploring alternative approaches to improve the management of BRAF^V600E^ in GBM.

RES (3,5,4’-trihydroxy-trans-stilbene) is a polyphenolic compound found in various natural sources [18]. It has been well documented that RES exerts inhibitory effects on a variety of cancers, including GBMs [19,20]. Moreover, the MAPK [21] and STAT3 signaling pathways are its major molecular targets [22]. Zhang et al. (2024) demonstrated that RES suppresses JAK2/STAT3 signaling and attenuates NLRP3 inflammasome activation in GBM, thereby remodeling the neuroinflammatory tumor microenvironment [23]. Furthermore, Wu et al. (2023) showed that RES enhances TMZ efficacy in GBM cells by downregulating MGMT expression through STAT3 inactivation via negative regulators PIAS3, SHP1, SHP2, and SOCS3, and notably reversed TMZ resistance in the LN428 cells [24]. A systematic review and meta-analysis by Luís et al. (2023) further confirmed that RES significantly reduced tumor volume in glioma xenograft models, with RES combined with TMZ demonstrating superior efficacy over TMZ monotherapy, providing robust translational support for RES-based therapeutic strategies in GBM [25]. Collectively, these findings highlight the multi-pathway antitumor potential of RES and support its further investigation as a therapeutic agent in molecularly stratified GBM [26]. We hypothesize that pharmacological inhibition of the BRAF/MAPK pathway by D+T induces a compensatory activation of STAT3 signaling that promotes GBM cell survival and therapeutic resistance. Furthermore, we propose that RES enhances therapeutic efficacy by suppressing this STAT3-dependent adaptive feedback mechanism. Accordingly, we investigated whether RES could suppress this STAT3-dependent adaptive feedback and improve therapeutic responsiveness in GBM cell lines with and without the BRAF^V600E^ mutation.

## 2. Material and Methods

### 2.1. GBM Cell Lines and Cell Culture

The U251 GBM cell line was sourced from the Cell Bank of the Chinese Academy of Sciences (Shanghai, China) and authenticated by STR profiling. The LN428 cell line was supplied by Professor Nicolas de Tribolet (University of Lausanne, Lausanne, Switzerland) and verified for BRAF^V600E^ mutation status by Sanger sequencing, which confirmed its reported genetic profile. The anaplastic thyroid cell line THJ-21T was employed as a positive BRAF^V600E^ control [27], and was provided by Professor Qiang Liu (Dalian Medical University, Dalian, China) with authorization from the Mayo Clinic Medical Education Research Foundation, USA. The cell lines were cultured in high-glucose Dulbecco’s Modified Eagle Medium (DMEM; Gibco, Grand Island, NY, USA) containing 10% fetal bovine serum (FBS) and 1% penicillin–streptomycin, and maintained at 37 °C in a humidified atmosphere containing 5% CO_2_.

### 2.2. Cell Treatments

RES (Sigma-Aldrich, St. Louis, MO, USA), dabrafenib (HY-14660; MCE, Monmouth Junction, NJ, USA), and trametinib (HY-10999; MCE, Monmouth Junction, NJ, USA) were dissolved in dimethyl sulfoxide (DMSO; Sigma-Aldrich) to prepare stock solutions at concentrations of 100 mM, 0.5 mM, and 0.5 mM, respectively. These stock solutions were subsequently diluted in a culture medium to final working concentrations of dabrafenib (0.5 µM) [28], trametinib 0.5µM [29], and RES 100µM [30], either alone or in combination, for all experiments. The compounds were administered for 48 h, and coverslips containing cells were then fixed with cold acetone or 4% paraformaldehyde (PFA; G1101, Servicebio, Wuhan, China) for the haematoxylin and eosin (H&E), immunofluorescence (IF), and immunocytochemistry (ICC) experiments. To ensure accuracy, each experiment was performed in triplicate.

### 2.3. Cell Counting Kit-8 (CCK-8) Assay

Cell proliferation was evaluated using the Cell Counting Kit-8 according to the manufacturer’s instructions. GBM cells were seeded in 96-well plates at a density of 5 × 10^5^ cells per well and incubated for 24, 48, and 72 h. After adherence, the cells were treated with different concentrations of RES (25–200 µM), dabrafenib (0.25–1.00 µM), and trametinib (0.25–1.00 µM). After 2 h of incubation with CCK-8 reagent at 37 °C, absorbance was measured at 450 nm using a Multiskan GO spectrophotometer (Thermo Fisher Scientific, Waltham, MA, USA). Cell growth inhibition was calculated using the following formula: % Inhibition = [1 − (OD of the treated group/OD of the control group)] × 100%.

### 2.4. Haematoxylin and Eosin (H&E) Staining

The cells were cultured on glass coverslips (Biosharp Life Sciences, Hefei, China) in 6-well plates and subjected to H&E staining. Following treatment, the cells were incubated with a culture medium containing RES, D+T, or RES+D+T for 48 h. After treatment, the cells were washed three times with phosphate-buffered saline (PBS) and fixed in acetone for 5 min at room temperature, followed by three additional PBS washes. The nuclei were stained with hematoxylin, rinsed with double-distilled water, and counterstained with eosin after differentiation. The coverslips were then sequentially dehydrated using 75% and 95% ethanol, absolute ethanol, ethanol–xylene, and xylene. Finally, the cells were mounted with natural gum, and morphological features were observed and photographed under a light microscope.

### 2.5. Cell Migration Assay

Cell migration was assessed using Transwell chambers (LABSELECT, Hefei, China). The U251 and LN428 GBM cells were seeded into the upper chambers at a density of 4 × 10^5^ cells in 200 µL of serum-free medium and treated according to the experimental groups. The lower chambers were filled with 600 µL of DMEM supplemented with 10% FBS (Gibco, USA). After 48 h of incubation at 37 °C, non-migrated cells on the upper surface of the membrane were gently removed using cotton swabs. Migrated cells on the lower surface of the membrane were fixed with acetone and stained with 0.5% crystal violet. The number of migrated cells was counted in five randomly selected fields per membrane under a light microscope.

### 2.6. Cell Viability Assay

GBM cell viability after treatment with RES, D+T, and RES+D+T was evaluated using a Calcein AM/PI assay kit (Cat. No. C2015M; Beyotime, Shanghai, China). In 24-well plates, the LN428 and U251 cells were seeded and treated with RES, D+T, or RES+D+T for 48 h. Viability was calculated as follows: cell viability (%) = (number of Calcein-AM^+^ cells)/(number of Calcein-AM^+^ cells + number of PI^+^ cells) × 100. The results for each experimental group were examined using fluorescence microscopy. The experiment was performed in triplicate to ensure consistent findings.

### 2.7. EDU Cell Proliferation Assay

To assess drug-induced alterations in cellular proliferation, the LN428 and U251 cells were seeded into 48-well plates at a standardized density. Following 48 h of treatment with RES, D+T, or RES+D+T, the cells were fixed in ice-cold absolute ethanol for 10 min, washed three times with phosphate-buffered saline (PBS), and permeabilized with 0.3% Triton X-100 (ST797; Beyotime, China) for 15 min. EdU incorporation was detected using the BeyoClick™ EdU Cell Proliferation Kit (Beyotime Biotechnology, Shanghai), and fluorescence images were acquired using a high-resolution fluorescence microscope.

### 2.8. TUNEL Assay

Apoptosis in GBM cells following treatment with RES, D+T, or RES+D+T was evaluated via terminal deoxynucleotidyl transferase dUTP nick-end labeling (TUNEL) assay. The cells were cultured overnight on sterile glass coverslips (BioSharp Life Sciences, Hefei, China) placed in 6-well plates. After treatment, the cells were washed three times with PBS and permeabilized with 0.1% Triton X-100 for 10 min at room temperature. Apoptotic nuclei were labeled using a commercial TUNEL assay kit (Beyotime Biotechnology, Shanghai, China), which produces green fluorescence upon DNA fragmentation. Fluorescence signals were captured and digitally archived using an Axio Imager.Z2 fluorescence microscope (ZEISS, Oberkochen, Germany).

### 2.9. Immunocytochemistry (ICC) Staining

For ICC analysis, coverslips from each experimental group were incubated with primary antibodies diluted in blocking buffer: rabbit polyclonal anti-BRAF (1:50) and rabbit polyclonal anti-pERK (1:200). The coverslips were washed three times with PBS, then permeabilized with 0.2% Triton X-100 for 10 min. Endogenous peroxidase activity was quenched by incubation with Reagent-1 (endogenous peroxidase inhibitor; Beyotime) at 37 °C for 10 min. Primary antibodies were applied overnight at 4 °C. Signal detection was performed using 3,3′-diaminobenzidine tetrahydrochloride (DAB) as the chromogenic substrate, and images were acquired using a LEICA DMI4000B inverted microscope (Wetzlar, Germany).

### 2.10. Immunofluorescence (IF) Staining

The cells were cultured on sterile glass coverslips in 6-well plates and treated with RES, D+T, or RES+D+T for 48 h. Post-treatment, the coverslips were rinsed thoroughly with PBS, fixed in ice-cold acetone for 15 min, and permeabilized with 0.1% Triton X-100 for 10 min. Endogenous peroxidase activity was blocked with Reagent-1 (Beyotime) at 37 °C for 10 min. The coverslips were then incubated overnight at 4 °C with rabbit monoclonal anti-pSTAT3 antibody (diluted according to manufacturer specifications). After three PBS washes, Alexa Fluor 488- or 594-conjugated goat anti-rabbit IgG secondary antibody (1:200) was applied for 1 h in the dark. The nuclei were counterstained with Hoechst 33,342 or DAPI (5 min), and immunofluorescence images were acquired using an Axio Imager.Z2 microscope (ZEISS).

### 2.11. RT-PCR and Sanger Sequencing

To evaluate BRAF^V600E^ mutation status and expression levels in GBM cell lines, total RNA was extracted from the LN428, U251, and THJ-21T cells using TRIzol reagent (TaKaRa, Kusatsu, Japan), following the manufacturer’s instructions. RNA concentration and purity were determined using a NanoDrop spectrophotometer (Thermo Fisher Scientific, Waltham, MA, USA). Complementary DNA (cDNA) was synthesized from 1 µg of total RNA using the PrimeScript RT Master Mix (TaKaRa). Quantitative real-time PCR (RT-qPCR) was performed in triplicate on a CFX96 Real-Time System (Bio-Rad, Hercules, CA, USA) using Premix Ex Taq (TaKaRa), with β-actin serving as the endogenous reference gene. Primer sequences (designed and validated by Tsingke Biotechnology, Beijing, China) were as follows: β-actin forward, 5′-CTCCATCCTGGCCTCGCTGT-3′; reverse, 5′-GCTGTCACCTTCACCGTTCC-3′; BRAF exon 15 forward, 5′-TCATAATGCTTGCTCTGATAGGA-3′; reverse, 5′-GGCCAAAAATTTAATCAGTGGA-3′. Amplification products (5 µL) were resolved on a 3.0% agarose gel containing ethidium bromide (0.5 µg/mL) and visualized under UV light using a Jim-X Scientific transilluminator (Shanghai, China). Remaining PCR amplicons were purified and subjected to bidirectional Sanger sequencing (Tsingke Biotechnology); sequence chromatograms were analyzed using ChromasPro, (version 2.1.10) software to confirm BRAF^V600E^ status.

### 2.12. Protein Preparation and Western Blot Analysis

Following treatment with RES, D+T, or RES+D+T, the cells were lysed on ice for 30 min in RIPA lysis buffer supplemented with protease and phosphatase inhibitor cocktails (Beyotime, Shanghai, China). The lysates were centrifuged at 12,000× *g* for 15 min at 4 °C, and supernatants were collected. Protein concentration was determined using the BCA Protein Assay Kit (Beyotime). Equal amounts of protein (20–40 µg) were resolved by SDS-PAGE on 10% polyacrylamide gels (G2177-50T; Servicebio), followed by electrophoretic transfer onto PVDF membranes (Millipore, Burlington, MA, USA). The membranes were blocked with 5% non-fat dry milk in TBST for 2 h at room temperature, washed three times with TBST (8 min each), and incubated overnight at 4 °C with primary antibodies: rabbit anti-pSTAT3 (1:800; D155018, BBI Life Sciences, Shanghai, China), rabbit anti-BRAF (1:1000; 53968, SAB, Riyadh, Saudi Arabia), rabbit anti-pERK (1:800; AF1015, Affinity Biosciences, Cincinnati, OH, USA), rabbit anti-BAX (1:1000; 50599-2-lg, Proteintech), rabbit anti-BCL-2 (1:1000; 26593-1-AP, Proteintech, Rosemont, IL, USA), and rabbit anti-GAPDH (1:5000; 10494-1-AP, Proteintech). After three TBST washes, membranes were incubated with HRP-conjugated goat anti-rabbit IgG secondary antibody (1:3000; SE134, Solarbio) for 1 h at room temperature. Immunoreactive bands were visualized using enhanced chemiluminescence (ECL) reagent (Servicebio) and captured using a ChemiDoc MP Imaging System (Bio-Rad). Band intensities were quantified using ImageJ software (version 154.p), normalized to GAPDH, and expressed relative to the untreated controls.

### 2.13. Statistical Analysis

Quantitative data are presented as mean ± standard deviation (SD) from at least three independent biological replicates. Statistical significance was determined by one-way ANOVA followed by Dunnett’s post hoc test. A *p*-value < 0.05 was considered statistically significant. All the analyses were performed using GraphPad Prism version 9.0.

## 3. Results

### 3.1. BRAF^V600E^ Point Mutation in LN428 Cells

**Figure 1:**
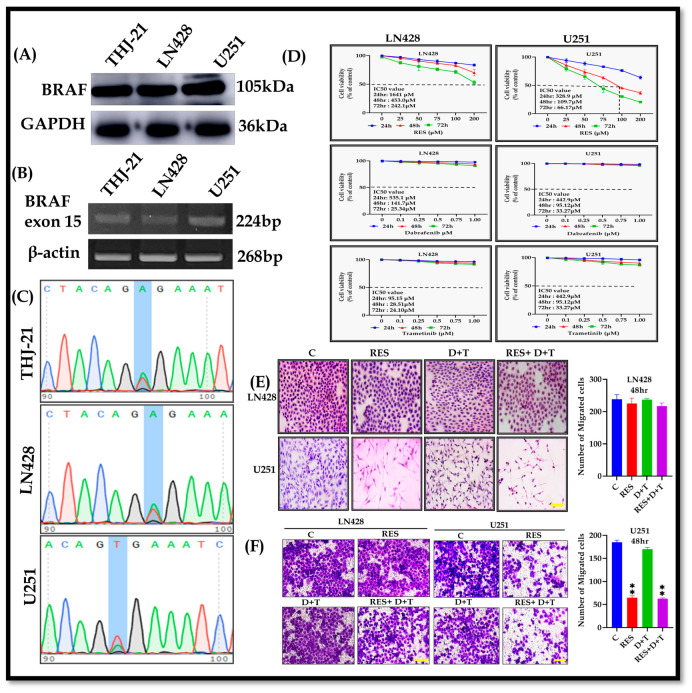
BRAF expression, mutation status, and therapeutic response of GBM cells to RES and BRAF/MEK inhibitors. (**A**) Western blot analysis of BRAF protein expression in THJ-21, LN428, and U251 cells. GAPDH serves as loading control. (**B**) RT-PCR amplification of BRAF exon 15 with β-actin as internal control. (**C**) Sanger sequencing chromatograms of BRAF exon 15. Highlighted nucleotide indicates mutation site aligned with reference sequence NM_004333. (**D**) Concentration–response curves of LN428 and U251 cells treated with RES, 20 dabrafenib, or trametinib at 24, 48, and 72 h assessed by CCK-8 assay. (**E**) H&E staining of LN428 and U251 cells treated with RES, D+T, or RES+D+T. (**F**) Transwell migration assay of both cells treated with RES, D+T, or RES+D+T. Data are presented as mean ± SD from three independent experiments. Statistical analysis by one-way ANOVA with Dunnett’s post hoc test. ** *p* < 0.01. Scale bar 100 µm.

To determine the BRAF^V600E^ mutational status in GBM cell lines, RT-PCR base Sanger sequencing was performed on the U251 and LN428 cells. Western blot analysis confirmed BRAF protein expression in both cell lines (Figure 1A). RNA isolation from the LN428 and U251 cells was subjected to BRAF exon 15-specific RT-PCR amplification, with the THJ-21T anaplastic thyroid cancer cells serving as a BRAF^V600E^-positive control [27]. RT-PCR analysis confirmed the BRAF gene was expressed in the LN428, U251, and THJ-21T cell lines (Figure 1B). Sanger sequencing revealed that the LN428 cell lines harbored a BRAF^V600E^ point mutation, characterized by a glutamate-to-valine substitution at codon 600 (Val600Glu) in exon 15 of the BRAF kinase domain. In contrast, the U251 cell line lacked this mutation (Figure 1C).

### 3.2. Different Response of LN428 and U251 Cells to the Drugs

To evaluate the anti-GBM efficacy of RES, dabrafenib, and trametinib, we assessed call viability using a CCK-8 assay across multiple concentrations and time points. RES treatment reduced the U251 cells’ viability in both dose-dependent (25, 50, 75, 100, and 200 µM) and time-dependent (24, 48, and 72 h) manners, with a calculated 50% inhibitory concentration (IC_50_) of 109.7 µM at 48 h. In contrast the LN428 cells demonstrated substantially reduced sensitivity to RES, exhibiting an IC_50_ of 453.1 µM—approximately 4.1-fold higher than the U251 cells. Similarly, dabrafenib induced dose-dependent (0.1, 0.25, 0.5, 0.75, and 1.0 µM) and time-dependent growth inhibition, yielding IC_50_ values of 141.7 µM in LN428 and 95.12 µM in the U251c cells. Trametinib exhibited comparable patterns with IC_50_ values of 28.51 µM and 20.01 µM in the LN428 and U251 cells, respectively (Figure 1D). Notably, the high IC_50_ values suggest intrinsic resistance to BRAF/MEK inhibition in both cell lines. The findings revealed that the LN428 cells are less sensitive to RES and D+T than the U251 cell line, highlighting the different degrees of sensitivity to chemotherapy between the two cell lines. Morphological assessment via H&E staining corroborated these viability findings. Following 48 h treatment, RES monotherapy and the RES+D+T combination induced pronounced cytoplasmic condensation and substantial reduction in cell density in the U251 cells, whereas there are no such effects observed in the LN428 cells. Notably, D+T combination therapy failed to elicit significant morphological alterations in either cell lines (Figure 1E).

### 3.3. RES Inhibits Migration in U251 but Not LN428 Cells

Transwell migration assays demonstrated that RES monotherapy and the RES+D+T combination significantly suppressed cell migration in the U251 cells compared to the controls (** *p* < 0.01). The LN428 cells showed no significant changes in migratory capacity under the same treatment conditions. D+T combination therapy failed to inhibit migration in both cell lines (Figure 1F).

### 3.4. Proliferation Is Suppressed by RES in U251 Cells

**Figure 2:**
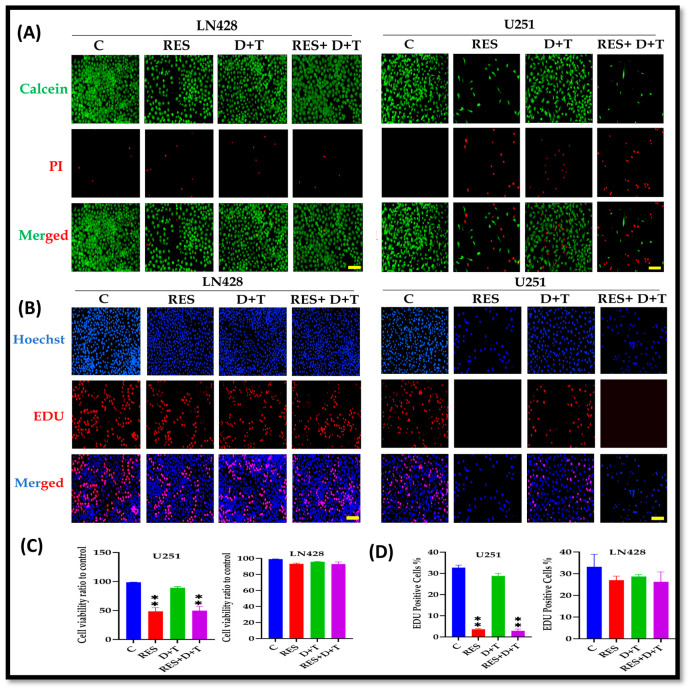
Differential cytotoxic and anti-proliferative effects of RES and BRAF/MEK inhibitors in GBM cells. (**A**) Representative fluorescence results of Calcein AM/PI dual staining in LN428 and U251 cells following 48 h treatment with RES, D+T, or RES+D+T. Green fluorescence indicates viable cells (Calcein AM-positive), red fluorescence indicates nonviable cells (PI-positive), and merged indicate overlay. (**B**) Representative fluorescence results of EdU incorporation assay in LN428 and U251 cells treated with RES, D+T, or RES+D+T. Blue fluorescence indicates nuclei (Hoechst), red fluorescence indicates proliferating cells (EdU-positive), and merged show overlay. (**C**) Quantification of cell viability ratio relative to control in LN428 and U251 cells. (**D**) Quantification of EdU-positive cells expressed as percentage of total cells in LN428 and U251 cells. U251 cells exhibited significant reductions in viability and proliferation following treatment with RES alone or RES+D+T compared to control, whereas LN428 cells demonstrated resistance to all treatments. Data are presented as mean ± SD from three independent experiments. Statistical analysis by one-way ANOVA with Dunnett’s post hoc test. ** *p* < 0.01. Scale bar 100 µm.

Calcein AM/PI dual staining revealed that RES monotherapy and the RES+D+T combination effectively reduced viable cell populations (green fluorescence) while increasing nonviable cells (red fluorescence) in the U251 cells. In contrast, the LN428 cells maintained high viability with predominantly Calcein AM-positive staining across all treatment groups, including D+T (Figure 2A,C). EdU incorporation assays showed that the LN428 cells exhibited significantly higher proportions of Edu-positive nuclei compared to the U251 cells following treatment with RES, D+T, or RES+D+T, indicating sustained DNA synthesis and proliferative activity. The U251 cells showed markedly reduced EdU-positive populations following RES alone or RES+D+T treatment (** *p* < 0.01). D+T combination therapy did not significantly suppress EdU incorporation in either cell line (Figure 2B,D).

### 3.5. RES Induces Apoptosis Selectively in U251 Cells

**Figure 3:**
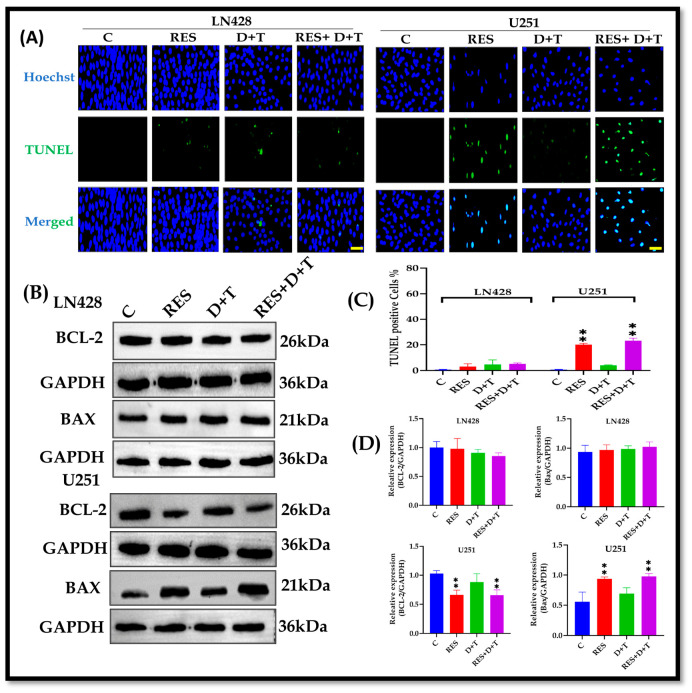
RES and RES+D+T induce apoptosis in U251 cells but not in LN428 cells. (**A**) TUNEL assay in LN428 and U251 cells following 48 h treatment with RES, D+T, or RES+D+T. (**B**) Western blot analysis showing BCL-2 and BAX protein expression in both cell lines treated with RES, D+T, or RES+D+T. (**C**) Quantification of TUNEL-positive cells expressed as percentage of total cells in LN428 and U251 cells. (**D**) Densitometric quantification of BCL-2 and BAX protein expression levels normalized to GAPDH in LN428 and U251 cells. U251 cells exhibited significant increase in apoptosis following treatment with RES alone or RES+D+T, although LN428 cells showed no significant apoptotic response to any treatment. Data are presented as mean ± SD from three independent experiments. Statistical analysis by one-way ANOVA with Dunnett’s post hoc test. ** *p* < 0.01. Scale bar 50 µm.

TUNEL assays revealed extensive TUNEL-positive nuclei (green fluorescence) in the U251 cells following RES or RES+D+T treatment. A quantitative analysis showed apoptosis rates of 4.7% in the LN428 cells and 4.0% in the U251 cells following D+T treatment, comparable to the untreated controls. RES significantly elevated apoptosis in the U251 cells, while the LN428 cells remained refractory across all treatments (Figure 3 A,C). A Western blot analysis of apoptosis-related proteins revealed that RES treatment, either alone or combined with D+T, significantly decreased BCL-2 expression while increasing BAX protein levels in the U251 cells (** *p* < 0.01). The D+T combination did not significantly modulate BCL-2 or BAX expression in either cell line. Neither RES monotherapy nor the RES+D+T combination induced significant alterations in BCL-2 or BAX expression in the LN428 cells (Figure 3B,D).

### 3.6. BRAF Expression Remains Unchanged While pERK Is Downregulated by RES

**Figure 4:**
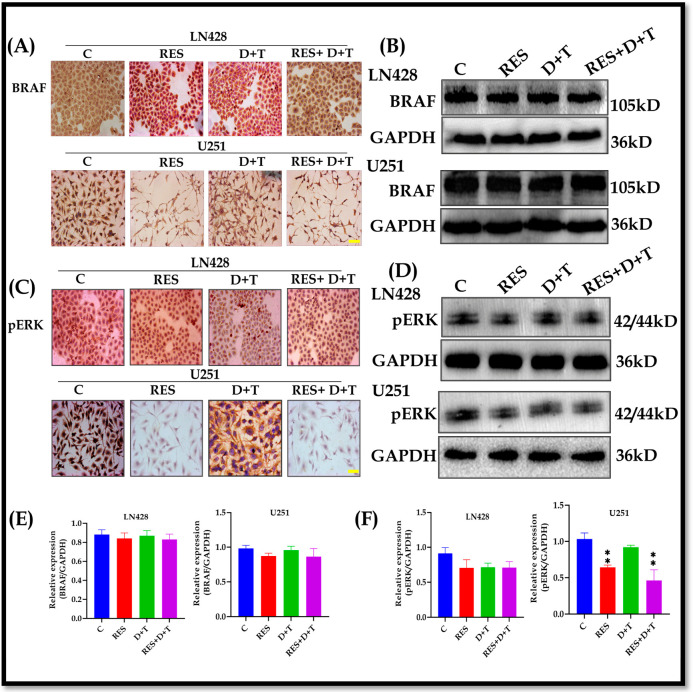
Differential effects of RES and BRAF/MEK inhibitors on BRAF and pERK expression in GBM cells. (**A**) ICC results of BRAF protein expression in LN428 and U251 cells treated with RES, D+T, or RES+D+T. (**B**) Western blot analysis of BRAF expression in LN428 and U251 cells. (**C**) ICC results of pERK protein expression in LN428 and U251 cells after treatment. (**D**) Western blot analysis of pERK protein expression. (**E**,**F**) Quantification of BRAF and pERK protein expression normalized to GAPDH. BRAF protein expression remained unchanged in both cell lines across all treatments. U251 cells results showed significant pERK reduction with RES or RES+D+T, while LN428 cells exhibited no significant changes. Data are presented as mean ± SD from three independent experiments. Statistical analysis by one-way ANOVA with Dunnett’s post hoc test. ** *p* < 0.01. Scale bar 50 µm.

ICC and Western blot analyses demonstrated that RES, D+T, or RES+D+T treatments did not significantly alter the BRAF protein expression levels in either the LN428 or U251 cells (Figure 4A,B,E). In contrast, ICC showed intense pERK staining (brown DAB precipitate) in the untreated LN428 and U251 cells. RES administration significantly reduced pERK immunoreactivity in the U251 cells in both monotherapy and RES+D+T groups. The Western blot results/quantification confirmed significant pERK downregulation in the RES-treated U251 cells (** *p* < 0.01). D+T combination therapy produced modest, statistically insignificant reductions in pERK levels in both cell lines. LN428 cells demonstrated resistance to pERK downregulation across all treatment groups (Figure 4C,D,F).

### 3.7. RES Suppresses D+T-Induced STAT3 Hyperactivation

**Figure 5:**
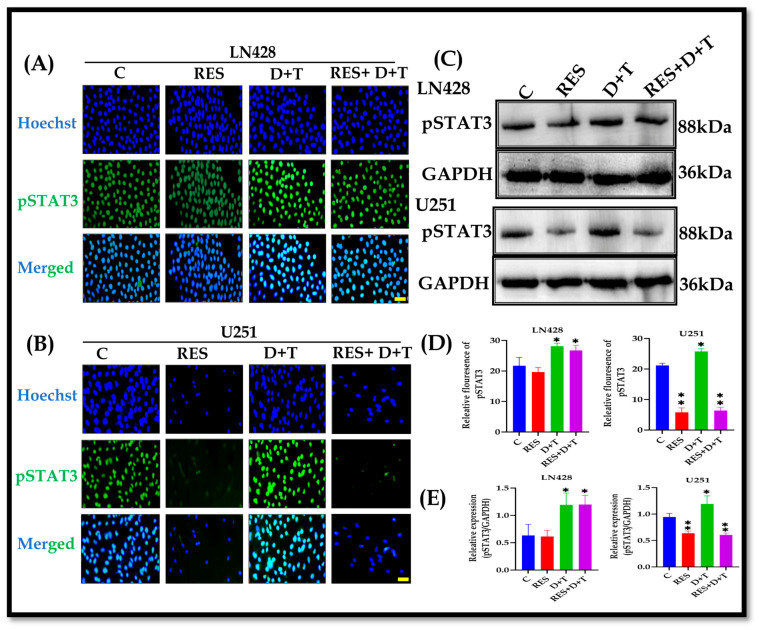
Differential modulation of pSTAT3 expression by RES and BRAF/MEK inhibitors in GBM cells. (**A**,**B**) IF staining showed pSTAT3 protein expression after treatment in LN428 and U251 cells. (**C**) Western blot analysis of pSTAT3 expression in LN428 and U251 cells. (**D**) Quantification of pSTAT3 fluorescence intensity in LN428 and U251 cells. (**E**) Densitometric quantification of pSTAT3 expression normalized to GAPDH in LN428 and U251 cells. RES either alone or in combination with D+T exhibited significant reduction in pSTAT3 protein levels in U251 cells, while LN428 cells showed no significant response to these treatments. D+T treatment significantly enhanced pSTAT3 expression in both cell lines. Data are presented as mean ± SD from three independent experiments. Statistical analysis by one-way ANOVA with Dunnett’s post hoc test. * *p* < 0.05, ** *p* < 0.01. Scale bar 50 µm.

IF staining revealed the predominantly nuclear localization of pSTAT3 in both cell lines under basal conditions. D+T combination therapy significantly enhanced pSTAT3 immunofluorescence intensity in both the LN428 and U251 cells relative to the controls. RES treatment significantly reduced pSTAT3 immunofluorescence in the U251 cells in both monotherapy and RES+D+T groups, effectively counteracting D+T-induced STAT3 hyperactivation. RES failed to downregulate pSTAT3 expression in the LN428 cells, even when combined with D+T (Figure 5A,B,D). The Western blot analysis further confirmed that D+T treatment significantly upregulated pSTAT3 protein expression in both the LN428 and U251 cells (* *p* < 0.05 or ** *p* < 0.01). RES monotherapy significantly suppressed pSTAT3 expression in the U251 cells, and the RES+D+T combination maintained this suppression despite the presence of D+T. In the LN428 cells, pSTAT3 expression remained elevated following all treatment groups, with neither RES alone nor the RES+D+T combination achieving significant downregulation (Figure 5C,E).

## 4. Discussion

GBM is recognized as the most aggressive form of brain tumor. The standard therapeutic approach generally involves surgical resection, followed by radiation therapy and chemotherapy with TMZ. However, a significant proportion of patients often face challenges due to the widespread resistance to radiation and chemotherapy [31]. Current therapeutic strategies often lack efficacy due to several factors, including genetic heterogeneity [2]. BRAF gene mutations are found in various cancers including GBM [32], and the treatment responses remain unclear in glioma [33]. Our previous research showed a significantly downregulated expression of the BRAF gene with the BRAF^V600E^ mutation in anaplastic thyroid cancer [27]. Therefore, the finding of BRAF^V600E^ point mutation among GBM cases brings hope for RES- and RES+D+T-based targeting therapy. In this study, we checked the presence of BRAF^V600E^ in RES-sensitive U251 and RES-resistant LN428 GBM cells by Sanger sequencing, using the human anaplastic thyroid cancer cell line THJ-21 as a BRAF^V600E^-positive control [27]. The results confirmed the presence of the BRAF^V600E^ point mutation in the LN428 cell line, while this mutation was absent in the U251 cell line. The differences in RES sensitivity and BRAF genotypic status between these two GBM cell lines make them ideal experimental models for exploring the management of drug-resistant GBM cells.

In this study, we investigated the efficacy of RES, D+T, and their combination against U251 cells without and LN428 cells with the BRAF^V600E^ mutation. RES significantly reduced U251 cell viability in a time- and dose-dependent manner. In contrast, the LN428 cells demonstrated markedly lower sensitivity to RES, suggesting the need for alternative treatment strategies. Moreover, both cell lines responded poorly to the D+T combination, as indicated by their high IC_50_ values. For instance, the combination of RES+D+T resulted in similar inhibitory effects on the U251 cells as RES monotherapy, suggesting that the suppressive effects resulted from RES rather than dabrafenib/trametinib. In other words, the above results are reasonable because the U251 cells are free of the BRAF^V600E^ mutation. In the LN428 cells, neither RES, nor D+T, nor their combination caused cell suppression. In addition, BAX and BCL-2 play opposing roles in determining cell survival or death [34]. We found that RES enhanced pro-apoptotic BAX expression and reduced anti-apoptotic BCL-2 expression in the U251 cells; in contrast, no significant changes in BAX and BCL-2 expression were observed in the LN428 cells, irrespective of drug treatment. These findings highlight the complexity of GBM cell responses to chemotherapy and the need for further exploration of their molecular heterogeneity.

The MAPK signaling pathway has been identified as a critical factor influencing cancer drug sensitivity [35], with MAPK signaling-associated genes, such as mutant BRAF and NF1, playing key roles in cancer formation and progression [36]. Sanger sequencing confirmed the presence of the BRAF^V600E^ mutation in the LN428 cells but not in the U251 cells, underscoring the molecular heterogeneity of GBMs. Our Western blot and immunocytochemistry analyses revealed that BRAF protein expression was not downregulated in either cell line following treatment with RES monotherapy or the D+T combination. Although the D+T combination has been documented to inhibit the MAPK pathway through dual mechanisms—dabrafenib targeting BRAF and trametinib targeting MEK—and to suppress the proliferation of BRAF^V600E^ mutant cells with enhanced antitumor efficacy [15], our comprehensive analysis revealed that treatment with the D+T combination did not elicit a significant response in either the BRAF^V600E^ mutant or wild-type cell lines. This observation contrasts with the established efficacy of D+T in BRAF^V600E^ mutant melanoma [37]. Chen and Park (2023) comprehensively reviewed tumor cell resistance to BRAF and MEK1/2 inhibition, identifying multiple adaptive responses, including paradoxical MAPK pathway reactivation via CRAF dimerization [38]. Moreover, the D+T combination is not always effective against BRAFV600E-harboring tumors and may be circumvented through alternative signaling, metabolic, or regulatory networks [38,39,40]. Importantly, RES significantly decreased pERK protein expression in the U251 cells, whereas the LN428 cells exhibited no significant response, further demonstrating resistance in BRAF^V600E^ mutant cells even to compounds targeting the MAPK pathway. Recent phosphoproteomic studies have revealed that the BRAF^V600E^ mutation induces fundamentally altered signaling networks compared to wild-type BRAF, with modified scaffolding protein interactions and feedback regulation mechanisms [41,42]. These results underscore the necessity for further investigation into the molecular mechanisms underlying resistance associated with the BRAF^V600E^ mutation in LN428 cells.

STAT3, a transcription factor located in the cytoplasm, is crucial for regulating cellular differentiation, proliferation, and immune responses. Its aberrant activation facilitates tumor growth and progression [43]. STAT3 activation is typically initiated by phosphorylation at tyrosine 705 (pY705) through various tyrosine kinases, including EGFR, Src, JAK, and ERK [44,45,46]. Furthermore, STAT3 activation is observed in GBM tumors and plays a significant role in GBM formation and progression [47]. Zhao et al. (2020) demonstrated that inhibition of the MAPK pathway in BRAF mutant melanoma cells directly activates the JAK2/STAT3 signaling, revealing a reciprocal crosstalk between these two pathways, and further showed that dual inhibition of both the MAPK and JAK2/STAT3 pathways is necessary to achieve meaningful tumor suppression in BRAF mutant tumors [48]. This mechanistic crosstalk explains the paradoxical pSTAT3 upregulation observed following D+T treatment in our study. Moreover, our previous study demonstrated that targeting BRAF^V600E^ with the D+T combination significantly elevated pSTAT3 expression in anaplastic thyroid cancer cells [27]. Therefore, it was essential to verify this possibility in GBM cells, as STAT3 activation is critical for GBM cells, and D+T has been used to treat GBMs with and without BRAF mutations. Our results showed that pSTAT3 protein expression was significantly upregulated following D+T treatment in both GBM cell lines and was downregulated upon RES supplementation in the U251 cells. These findings illustrate the dual inhibitory effects of RES on both the MAPK and STAT3 pathways. Consistent with this, Capogiri M et al. (2023) demonstrated that chronic BRAF inhibitor exposure in BRAF^V600E^ glioma models activates compensatory pro-survival pathways including AKT/mTOR, with EGFR feedback signaling identified as a principal escape mechanism, further reinforcing the inevitability of adaptive signaling feedback following MAPK-targeted therapy in BRAFV600E-driven tumors [40]. Comparative mechanistic evidence from structurally related flavonoid compounds further supports this multi-pathway framework; Rana et al. (2025) demonstrated that isorhamnetin, a 3’-0- methylated flavonol, simultaneously targets the PI3K/AKT, MAPK, and STAT3 signaling pathways, induces apoptosis via BAX/BCL2 modulation and caspase activation, suppresses metastasis through MMP-2/9 downregulation, and regulates redox signaling via Nrf2/HO-1 activation, collectively reinforcing the concept that flavonoid-class polyphenols function as multi-pathway inhibitors with broad antitumor activity across diverse cancer models [49]. However, in the LN428 cells, RES failed to downregulate pSTAT3 despite its dual inhibitory mechanism, suggesting that BRAF^V600E^ mutant cells possess resistance mechanisms independent of both the MAPK and STAT3 pathways. These findings establish LN428 as a valuable model for investigating BRAFV600E-associated therapeutic resistance and underscore the importance of compensatory signaling in developing effective GBM treatment strategies. This study has limitations, including unexplored upstream STAT3 activation mechanisms and the use of only two cell lines without in vivo/knockin/knockout validation, which may not fully capture the complex tumor microenvironment and clinical heterogeneity of GBM. Furthermore, alternative resistance mechanisms in LN428 cells beyond the BRAF/MAPK and STAT3 pathways remain to be elucidated. Nonetheless, our findings identify compensatory STAT3 pathway activation as a therapeutically relevant resistance mechanism during BRAF/MEK inhibition, warranting systematic future validation.

## 5. Conclusions

In conclusion, BRAF^V600E^-mutantional status critically determines RES therapeutic efficacy in GBM. RES monotherapy effectively suppressed MAPK and STAT3 signaling and inhibited proliferation, migration, and induced apoptosis in BRAF wild-type cells, while demonstrating no significant activity is BRAF^V600E^ mutant cells. Furthermore, D+T paradoxically upregulated pSTAT3 in both cell lines, an effect reversed by RES in the U251 cells, suggesting that BRAF^V600E^-driven resistance operates through alternative signaling mechanisms beyond the MAPK/STAT3 pathways. Collectively, these findings establish RES as a mutation-stratified dual MAPK/STAT3 inhibitor, highlighting BRAF mutational profiling as a critical prerequisite for optimizing therapeutic strategies in GBM.

## Data Availability

Data will be made available upon request.

## Abbreviations

GBM	Glioblastoma
RES	Resveratrol
D+T	Dabrafenib/trametinib
H&E	Haematoxylin and eosin
IF	Immunofluorescence
ICC	Immunocytochemistry
CCK-8	Cell Counting Kit-8
ANOVA	One-way analysis of variance
FBS	Fetal bovine serum
BRAF	V-raf murine sarcoma viral oncogene homolog B
MAPK	Mitogen-activated protein kinase
STAT3	Signal transducer and activator of transcription 3
TMZ	Temozolomide
SD	Standard deviation
TBS-T	Tris-buffered saline
